# The Effect of a Novel Highly Selective Inhibitor of the Sodium/Calcium Exchanger (NCX) on Cardiac Arrhythmias in *In Vitro* and *In Vivo* Experiments

**DOI:** 10.1371/journal.pone.0166041

**Published:** 2016-11-10

**Authors:** Zsófia Kohajda, Nikolett Farkas-Morvay, Norbert Jost, Norbert Nagy, Amir Geramipour, András Horváth, Richárd S. Varga, Tibor Hornyik, Claudia Corici, Károly Acsai, Balázs Horváth, János Prorok, Balázs Ördög, Szilvia Déri, Dániel Tóth, Jouko Levijoki, Piero Pollesello, Tuula Koskelainen, Leena Otsomaa, András Tóth, István Baczkó, István Leprán, Péter P. Nánási, Julius Gy Papp, András Varró, László Virág

**Affiliations:** 1 Department of Pharmacology and Pharmacotherapy, Faculty of Medicine, University of Szeged, Szeged, Hungary; 2 MTA-SZTE Research Group of Cardiovascular Pharmacology, Hungarian Academy of Sciences, Szeged, Hungary; 3 Department of Pathophysiology, "Victor Babes" University of Medicine and Pharmacy, Timisoara, Romania; 4 Department of Physiology, Faculty of Medicine, University of Debrecen, Debrecen, Hungary; 5 Department of Dental Physiology and Pharmacology, Faculty of Dentistry, University of Debrecen, Debrecen, Hungary; 6 Orion Pharma, Espoo, Finland; The Ohio State University, UNITED STATES

## Abstract

**Background:**

In this study the effects of a new, highly selective sodium-calcium exchanger (NCX) inhibitor, ORM-10962 were investigated on cardiac NCX current, Ca^2+^ transients, cell shortening and in experimental arrhythmias. The level of selectivity of the novel inhibitor on several major transmembrane ion currents (L-type Ca^2+^ current, major repolarizing K^+^ currents, late Na^+^ current, Na^+^/K^+^ pump current) was also determined.

**Methods:**

Ion currents in single dog ventricular cells (cardiac myocytes; CM), and action potentials in dog cardiac multicellular preparations were recorded utilizing the whole-cell patch clamp and standard microelectrode techniques, respectively. Ca^2+^ transients and cell shortening were measured in fluorescent dye loaded isolated dog myocytes. Antiarrhythmic effects of ORM-10962 were studied in anesthetized ouabain (10 μg/kg/min *i*.*v*.) pretreated guinea pigs and in ischemia-reperfusion models (I/R) of anesthetized coronary artery occluded rats and Langendorff perfused guinea pigs hearts.

**Results:**

ORM-10962 significantly reduced the inward/outward NCX currents with estimated EC50 values of 55/67 nM, respectively. The compound, even at a high concentration of 1 μM, did not modify significantly the magnitude of I_CaL_ in CMs, neither had any apparent influence on the inward rectifier, transient outward, the rapid and slow components of the delayed rectifier potassium currents, the late and peak sodium and Na^+^/K^+^ pump currents. NCX inhibition exerted moderate positive inotropic effect under normal condition, negative inotropy when reverse, and further positive inotropic effect when forward mode was facilitated. In dog Purkinje fibres 1 μM ORM-10962 decreased the amplitude of digoxin induced delayed afterdepolarizations (DADs). Pre-treatment with 0.3 mg/kg ORM-10962 (*i*.*v*.) 10 min before starting ouabain infusion significantly delayed the development and recurrence of ventricular extrasystoles (by about 50%) or ventricular tachycardia (by about 30%) in anesthetized guinea pigs. On the contrary, ORM-10962 pre-treatment had no apparent influence on the time of onset or the severity of I/R induced arrhythmias in anesthetized rats and in Langendorff perfused guinea-pig hearts.

**Conclusions:**

The present study provides strong evidence for a high efficacy and selectivity of the NCX-inhibitory effect of ORM-10962. Selective NCX inhibition can exert positive as well as negative inotropic effect depending on the actual operation mode of NCX. Selective NCX blockade may contribute to the prevention of DAD based arrhythmogenesis, *in vivo*, however, its effect on I/R induced arrhythmias is still uncertain.

## Introduction

Cardiac action potential is generated by simultaneous opening and closing of a wide range of transmembrane ion channels, as well as the activity of a number of electrogenic ion transport mechanisms. One of the essential ion transport proteins is the sodium/calcium exchanger (NCX) [1,2,3,4] which is a major contributor to myocardial Ca^2+^ homeostasis [5,6,7,8,9,10]. When the membrane potential is more negative than its equilibrium potential, NCX extrudes Ca^2+^ to the extracellular space (“forward” transport mode). Since the removal of a single Ca^2+^ ion is paralleled with the uptake of three Na^+^ ions, the “forward” transport mode of the NCX results in a net inward (i.e. depolarizing) current. If the intracellular Na^+^ transiently rises and the Ca^2+^ level in the cell is still low and/or the membrane potential is depolarized, the NCX operates in its “reverse” transport mode: removing three Na^+^ ions from, uptaking one Ca^2+^ ion into the cell, generating net outward (i.e. repolarizing) current [3,5,11,12,13]. Consequently, during the full cardiac cycle, due to the dynamically changing membrane potential and intracellular Na^+^ and Ca^2+^ concentrations, the transmembrane current generated by the NCX may substantially contribute to an intracellular Ca^2+^-gain or loss, furthermore, due to its electrogenic nature, it can also modulate the depolarization and repolarization processes of the action potential [14].

Most previous studies aimed to demonstrate the antiarrhythmic effect of NCX inhibition were based on the use of KB-R7943 and SEA-0400 [4,15,16]. However, these compounds were shown to lack proper selectivity, since at the same concentration they efficiently inhibited NCX current they also significantly decreased other transmembrane ionic currents, including L-type Ca current (I_CaL_). This latter observation is especially important since Ca^2+^-overload generated arrhythmogenesis and pathological automaticity was shown to critically depend on transmembrane Ca^2+^ movement trough I_CaL_ [7].

Previously, we also reported that the commonly used NCX inhibitor, SEA-0400, significantly suppressed both delayed and early afterdepolarizations (DADs and EADs, respectively) in dog cardiac preparations [17]. However, the selectivity of SEA-0400 for NCX versus L-type calcium current (I_Ca_) was found to be rather limited [18,19,20]. Recently, similar results on EAD and DAD formation have been obtained with another novel NCX inhibitor, ORM-10103, shown to have improved selectivity for NCX compared to SEA-0400 [21]. Although these results reinforced the old assumption that NCX inhibition, indeed, can eliminate Ca^2+^ overload induced triggered arrhythmias, ORM-10103 was also found to exhibit some inhibitory effect on I_Kr_. Therefore, there was still no unambiguous experimental evidence regarding the effects of selective NCX inhibition on cellular Ca^2+^-handling and cardiac action potential, though such effects may have a major impact on both arrhythmogenic trigger and substrate via influencing cellular Ca^2+^ concentration and modulating membrane voltage changes.

Since due to the lack of selective NCX inhibitors, the question how NCX inhibition may affect cardiac arrhythmogenesis, Ca^2+^ handling and contraction could not be answered convincingly, an alternative approach, action potential modelling has been used in order to predict the putative effect of NCX inhibition on cardiac action potential waveform [22,23,24]. Since at the moment our electrophysiological knowledge is rather incomplete, any result of the modelling work needs direct experimental validation, especially regarding such highly complex transport systems as the NCX, critically depending on both the momentary membrane potential and intracellular Ca^2+^ and Na^+^ concentrations.

**Fig 1:**
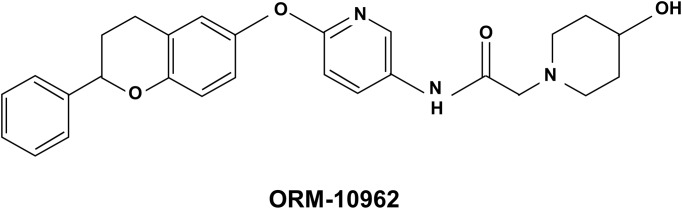
Chemical structure of ORM-10962 [2-(4-hydroxypiperidin-1-yl)-N-(6-((2-phenylchroman-6-yl)oxy)pyridin-3-yl)acetamide].

In an attempt to clarify these questions, in the present study we investigated the effects of a novel, highly selective and effective NCX inhibitor ORM-10962 ([2-(4-hydroxy-piperidin-1-yl)-N-(6-((2-phenylchroman-6yl)oxy)pyridin-3-yl)acetamide]; see Fig 1 for chemical structure) on the cardiac NCX current and Ca^2+^ handling, and also *in vivo* on ouabain or ischaemia induced cardiac arrhythmogenesis. Selectivity of the compound on L-type Ca^2+^ current, major repolarizing K^+^ currents, the late and peak sodium and Na^+^/K^+^ pump currents was also investigated.

## Materials and Methods

### Ethical statement

All experiments were carried out in compliance with the Guide for the Care and Use of Laboratory Animals (USA NIH publication No 85–23, revised 1996) and were approved by the Csongrád County Governmental Office for Food Safety and Animal Health, Hungary (approval No.: XIII/1211/2012). The ARRIVE guidelines were adhered to during the study, (NC3Rs Reporting Guidelines Working Group, 2010).

### Animals

Majority of measurements were performed in dog preparations. Guinea-pig papillary muscle preparations were used for slow response experiments. *In vivo* guinea pigs were used for ouabain induced arrhythmias and rats for coronary artery occlusion induced reperfusion induced arrhythmia studies. Guinea pigs were used for zero flow ischaemia study on Langendorff apparatus.

Mongrel dogs of either sex weighing 8 to 16 kg, and male Sprague-Dawley CFY rats weighing 290–330 g obtained from a licensed supplier were used for the study. The experimental animals were young adults.

### Housing and husbandry

Adult mongrel dogs exclusively bred for experimental purposes (age: 24–26 months, weight: 20–25 kg; supplied by the University of Szeged Experimental Animal Facility, 6781 Domaszék, Tanya 701., Hungary) were used for the study. The dogs were identified by a chip implanted subcutaneously in the neck area. All the dogs were under one roof and a common airspace, however, each dog had its own kennel space bordered by metal bars, so that animals could interact and see/hear each other while being safely separated. Each kennel had an “outdoor” area (about 4 x 2 m) still under a common roof of the animal house on the top level of the Department) and an indoor area (about 4 x 2 m) with central heating and a resting place, separated by a trapdoor. The animals were provided various types of chew toys for enrichment. A certified animal handler tended to the dogs every day (feeding, drinking, cleaning etc).

Male guinea-pigs (weighing 250–300 g) were kept in a separate room in groups of 6–10 in areas divided by large wooden boards on the floor covered with wooden chips for bedding. Each section had a surface area of 5 m^2^. Male Sprague-Dawley CFY rats weighing 290–330 g were maintained in standard rat cages. All the animal house was kept at standard temperature, humidity and lighting. The animals’ food intake and tap water consumption were provided *ad libitum*. The tap water is regularly checked for any pathogens.

### *In vitro* experiments

#### Voltage-clamp measurements

***Cell preparations*:** Ventricular myocytes were enzymatically dissociated from dog hearts by adapting a method described earlier in detail [21]. Following sedation of the dogs with xylazine (1 mg/kg, *i*.*v*.*)* and thiopental (30 mg/kg *i*.*v*.), each heart was rapidly removed through a right lateral thoracotomy and immediately rinsed in oxygenated modified Locke’s solution containing (in mM): NaCl 120, KCl 4, CaCl_2_ 1.0, MgCl_2_ 1, NaHCO_3_ 22, and glucose 11. The pH of this solution was set between 7.35 and 7.4 when saturated with the mixture of 95% O_2_ and 5% CO_2_ at 37°C One drop of cell suspension was placed in a transparent recording chamber mounted on the stage of an inverted microscope (Olympus IX51, Olympus, Tokyo, Japan), and individual myocytes were allowed to settle and adhere to the chamber bottom for at least 5–10 min before superfusion was initiated and maintained by gravity. Only rod-shaped cells with clear striations were used. HEPES-buffered Tyrode’s solution (composition in mM: NaCl 144, NaH_2_PO_4_ 0.4, KCl 4.0, CaCl_2_ 1.8, MgSO_4_ 0.53, glucose 5.5 and HEPES 5.0, at pH of 7.4) served as the normal superfusate.

***Recording procedures*:** Micropipettes were fabricated from borosilicate glass capillaries (Science Products GmbH, Hofheim, Germany), using a P-97 Flaming/Brown micropipette puller (Sutter Co, Novato, CA, USA), and had a resistance of 1.5–2.5 MOhm when filled with pipette solution (composition detailed later). The membrane currents were recorded with Axopatch-200B amplifiers (Molecular Devices, Sunnyvale, CA, USA) by means of the whole-cell configuration of the patch-clamp technique. The membrane currents were digitized with 250 kHz analogue to digital converters (Digidata 1440A, Molecular Devices, Sunnyvale, CA, USA) under software control (pClamp 10, Molecular Devices, Sunnyvale, CA, USA). Experiments were carried out at 37°C. For measuring the currents we applied relative similar voltage protocols as described earlier in details, however, many of them were partially adapted for focused reasons [21,25,26].

***Measurement of NCX current*:** For the measurement of the Na^+^/Ca^2+^ exchanger current (I_NCX_), the method of Hobai *et al* [27] was applied. Accordingly, the NCX current is defined as Ni^2+^-sensitive current and measured in a special K^+^-free solution (composition in mM: NaCl 135, CsCl 10, CaCl_2_ 1, MgCl_2_ 1, BaCl_2_ 0.2, NaH_2_PO_4_ 0.33, TEACl 10, HEPES 10, glucose 10 and ouabain 20 μM, nisoldipine 1 μM, and lidocaine 50 μM, at pH 7.4) as described earlier [21] in detail. The pipette solution used for recording I_NCX_ contained (in mM) CsOH 140, aspartic acid 75, TEACl 20, MgATP 5, HEPES 10, NaCl 20, EGTA 20 and CaCl_2_ 10, pH adjusted to 7.2 with CsOH.

***Measurement of L-type calcium current (I***_***CaL***_***)*:** The L-type calcium current (I_CaL_) was recorded in HEPES-buffered Tyrode’s solution supplemented with 3 mM 4-aminopyridine. A special solution was used to fill the micropipettes (composition in mM: KOH 40, KCl 110, TEACl 20, MgATP 5, EGTA 10, HEPES 10 and GTP 0.25, pH was adjusted to 7.2 by KOH).

***Measurement of potassium currents*:** The inward rectifier (I_K1_), transient outward (I_to_), rapid (I_Kr_) and slow (I_Ks_) delayed rectifier potassium currents were recorded in HEPES-buffered Tyrode’s solution. The composition of the pipette solution (in mM) was the following: KOH 110, KCl 40, K_2_ATP 5, MgCl_2_ 5, EGTA 5, HEPES 10 and GTP 0.1 (pH was adjusted to 7.2 by aspartic acid). 1 μM nisoldipine was added to the bath solution to block I_CaL_. When I_Kr_ was recorded, I_Ks_ was inhibited by using the selective I_Ks_ blocker HMR 1556 (0.5 μM). During I_Ks_ measurements, I_Kr_ was blocked by 0.5 μM dofetilide and the bath solution contained 0.1 μM forskolin.

Since the run-down of I_CaL_ and I_Ks_ currents is commonly seen during the measurements, the current level was monitored during the initial equilibration period and also at the end of the measurements when a washout period of at least 10 min was applied in order to draw a distinction between drug effect and rundown of the current. The cells, in which excessive rundown was observed, were omitted from the analysis.

***Measurement of late sodium current (I***_***NaL***_***)*:** The sodium current was activated by depolarizing voltage pulses to -20 mV from the holding potential of -120 mV. After 5–7 min incubation with ORM-10962 the external solution was replaced by that containing 20 μM TTX. TTX at this concentration completely blocks the late sodium current (I_NaL_). The external solution was HEPES-buffered Tyrode’s solution supplemented with 1 μM nisoldipine, 0.5 μM HMR-1556 and 0.1 μM dofetilide in order to block I_CaL_, I_Ks_ and I_Kr_ currents. The composition of the pipette solution (in mM) was: KOH 110, KCl 40, K_2_ATP 5, MgCl_2_ 5, EGTA 5, HEPES 10 and GTP 0.1 (pH was adjusted to 7.2 by aspartic acid).

All of the voltage clamp data that is presented is normalized to the capacity of the cell and is expressed as current density (pA/pF). When concentration-dependent effect of ORM-10962 on NCX current was measured the concentration of the drug was expressed as the log10 of concentration in Mol to follow the usual procedure.

***Measurement of peak sodium current (I***_***Na***_***) in CHO cells*:** cDNA clone of the cardiac sodium channel Nav1.5 was provided by György Panyi [28] and was heterologously over-expressed in Chinese hamster ovary cells (CHO; ATCC, Manassas, Virginia,USA). CHO cells were cultured in F12 medium (Lonza, Verniers, Belgium) supplemented with 10% fetal bovine serum (PAA, Paschling, Austria) at 37°C in a humidified atmosphere containing 5% CO_2_. Transient transfections were carried out as follows. CHO cells were plated one day before transfection in 60 mm diameter culture dishes. 2 μg of the Nav1.5 plasmid DNA, 1 μg green fluorescent protein (GFP) over-expressing plasmid and 20 μg polyethylenimine (25kDa, linear, Polysciences Inc., PA, USA) were combined in 1.5 ml serum-free F12. The transfection mixture was incubated at room temperature for 30 minutes, during which time the cells were washed with serum-free F12 twice. After washing, the transfection mixture was added to the cells and the culture dishes were moved back into the CO_2_ incubator for 2 hours. At the end of the incubation period, the transfection mixture was replaced by growth medium. Plasmid samples used in the transfections were purified by Nucleobond PC100 anion-exchange columns (Macherey-Nagel GmbH & Co. KG, Düren, Germany). 48 hours post-transfection the cells were briefly trypsinized, collected by centrifugation and were re-suspended in growth medium.

Transfected CHO cells were replaced in the bath and superfused with HEPES-buffered Tyrode’s solution. Inverted fluorescent microscope (Olympus IX51) allowed for visualisation of the GFP fluorescence. The electrodes were filled with the pipette solution (in mM): KOH 110, KCl 40, K_2_ATP 5, MgCl_2_ 5, EGTA 5, HEPES 10 and GTP 0.1 (pH was adjusted to 7.2 by aspartic acid). In GFP positive cells the sodium peak current was recorded at room temperature (~23°C). Capacitance of cells was cancelled and series resistance was compensated to 70–80%. Current was elicited by step pulse to -20mV for 20ms from the holding potential of -90mV.

***Measurement of Na***^**+**^***/K***^**+**^ ***pump current*:** Steady-state current at -30 mV was recorded in control conditions and in the presence of ORM-10962. After 5–7 min incubation with ORM-10962 the normal external solution (composition in mM: NaCl 135, CsCl 2, KCl 5, MgCl_2_ 1, CdCl_2_ 0.2, BaCl_2_ 2, HEPES 5, glucose 10, pH 7.4 by NaOH) was replaced by K^+^ free solution. The Na^+^/K^+^ pump current (I_p_) was defined as the difference between currents measured in 5mM K^+^ and in 0 mM K^+^ containing solutions. The composition of the pipette solution (in mM) was: CsOH 100, aspartic acid 100, NaCl 30, MgATP 5, MgCl_2_ 2, TEACl 20, EGTA 5, HEPES 10 and glucose 10 (pH was adjusted to 7.2 by CsOH).

#### Action potential measurements with standard microelectrode technique

***Recording of delayed afterdepolarizations*:** For measuring action potentials we applied relative similar protocols as described earlier in detail, however, many of them were partially adapted for focussed reasons [21,25,26].

The same adult mongrel dogs of either sex weighing 8–16 kg were used. Purkinje strands obtained from both ventricles and right ventricular papillary muscle tips were mounted individually in a tissue chamber superfused with oxygenated Locke’s solution at 37°C. Each preparation was stimulated (HSE stimulator type 215/II), initially at a constant cycle length of 1000 ms (frequency 1 Hz), with rectangular constant current pulses 2 ms in duration. The current pulses were isolated from ground and delivered through bipolar platinum electrodes in contact with the preparations. At least 1 h was allowed for each preparation to equilibrate during continuous superfusion with modified Locke’s solution, warmed to 37°C before the experimental measurements commenced. Transmembrane potentials were recorded with the use of conventional 5–20 MΩ, 3 M KCl-filled microelectrodes connected to the input of a high-impedance electrometer (Biologic Amplifier VF 102, Claix, France). The first derivative of transmembrane potential (dV/dt_max_) was obtained electronically with a Biologic DV-140 (Claix, France) differentiator designed and calibrated to give a linear response over the range between 10 and 1000 V/s. In each experiment, the baseline action potential characteristics were first determined during continuous pacing at 1 Hz (on Purkinje fibres, continuous pacing at 2 Hz), and then when the pacing cycle length was sequentially varied from 300 to 5000 ms. The 25^th^ action potential was measured at each cycle length, and the cycle length was then changed so that quasi-steady-state frequency-response relations could be generated rapidly. The preparations were next superfused with the drug for 40–60 min before the pacing protocol was repeated and the parameters were measured again. Efforts were made to maintain the same impalement throughout each experiment. If an impalement became dislodged, however, electrode adjustment was attempted and, if the action potential characteristics of the re-established impalement deviated by less than 5% from those of the previous measurement, the experiment was continued. When this 5% limit was exceeded, the experiment was terminated and all the data involved were excluded from the analyses.

***Recording of slow response action potentials*:** Adult guinea-pigs of either sex weighing 250–300 g were anticoagulated with sodium-heparin and anaesthetized with 30 mg/kg *i*.*v*. thiopental after sedation (xylazine, 1 mg/kg i.v.). The hearts were rapidly removed through a right lateral thoracotomy and immediately rinsed in ice-cold Krebs-Henseleit solution (containing in mM: NaCl 118.5, KCl 4.0, CaCl_2_ 2.0, MgSO_4_ 1.0, NaH_2_PO_4_ 1.2, NaHCO_3_ 25.0 and glucose 10.0). The pH of this solution was set to 7.35±0.05 when saturated with a mixture of 95% O_2_ and 5% CO_2_. After excision, the right or left ventricular papillary muscle preparations were immediately mounted in a 40 ml tissue chamber, and initially perfused with normal Krebs-Henseleit solution.

The preparations were stimulated (Experimetria Ltd, PW-01, Hungary) by means of constant rectangular voltage pulses (1 ms) delivered through a pair of bipolar platinum electrodes at a frequency of 1 Hz. Action potentials were recorded with a conventional microelectrode technique. Sharp microelectrodes, with a tip resistance of 10–20 MΩ when filled with 3 M KCl, were connected to the amplifier (Biologic Amplifier, model VF 102). The voltage output from the amplifier was sampled with an AD converter (NI 6025, Unisip Ltd, Hungary). The action potential amplitude and maximum rate of depolarization were obtained with Evokewave v1.49 software (Unisip Ltd, Hungary). Each preparation was allowed for at least 30 min to equilibrate in normal Krebs-Henseleit solution at 37°C.

Slow response action potentials were established in modified Krebs-Henseleit solution containing 25 mM KCl, supplemented with 100 μM BaCl_2_ to inhibit I_K1_ and with 1 μM forskolin to increase I_CaL_. dV/dt_max_ values in the interval 5–20 V/s, and amplitudes of at least 60 mV were accepted; data from experiments in which these levels were not met were discarded.

#### Measurements of [Ca^2+^]i transients and cell shortening

For measuring fluorescent optical signals, isolated dog ventricular myocytes were used loaded with a Ca^2+^ sensitive dye Fluo-4AM for 20 minutes in room temperature in dark. One drop of the loaded cells were placed in a cell chamber (RC47FSLP, Warner Instruments, Hamden, CT, USA) and were field stimulated at 1 Hz (PW-01, Experimetria Ltd. Hungary). The measurement was performed on an inverted microscope (Olympus IX 71; Olympus Cor-poration, Tokyo, Japan). The fluorescent dye was excited at 480 nm and emitted at 535 nm. Ca^2+^ signals were recorded by a photon counting photomultiplier module (Hamamatsu, model H7828; Hamamatsu Photonics Deutschland GmbH, Herrsching am Ammersee, Germany) and were digitized and sampled at 1 kHz by using Digidata 1440A interface (Axon Instruments). The data acquisition and analysis were performed by Clampex 10.0 and Clampfit 10.0 (Axon Instruments). The transient amplitudes were calculated as a difference of the peak and diastolic fluorescent values. Background fluorescence levels were used to correct raw fluorescence data. The Ca^2+^ transients were normalized to the diastolic fluorescent level. The cell shortening was measured by using a video edge detector system (VED-105, Crescent Electronics, Sandy, UT, USA).

The control transients were monitored in a normal HEPES-buffered Tyrode’s solution (containing in mM: 144 NaCl, 0.4 NaH_2_PO_4_, 4 KCl, 0.53 MgSO_4_, 1.8 CaCl_2_, 5.5 glucose and 5 HEPES; pH was adjusted to 7.4 with NaOH) then were switched to modified Tyrode’s solution containing 70 mM NaCl and 70 mM cholin-cloride or normal Tyrode’s solution supplemented with 600 nM forskolin depending on the experimental group. After recording the control transients, 1 μM ORM-10962 were added to the solution.

#### Statistical analysis

All data are expressed as means ± standard error (SEM). Statistical analysis was performed with Student’s *t-*test for paired data. The results were considered statistically significant when P was < 0.05.

### In vivo experiments

Male guinea pigs and Sprague-Dawley CFY rats were investigated in ouabain induced and ischaemia-reperfusion induced arrhythmia models, respectively. Animals were fed with standard chow for rodents (Szindbád Ltd., Gödöllő, Hungary) and were allowed to drink tap water *ad libitum*. 12h dark/light cycles were maintained.

The outcome of the mortality during the experiments was in agreement with that of anticipated such that it was reviewed and approved by the animal ethics committee as part of our approved protocol.

#### Ouabain induced arrhythmias

The methods described earlier by others [29,30] were applied. Male guinea-pigs, weighing 320-440g, were anaesthetized with pentobarbitone (45 mg/kg intraperitoneally). The trachea was prepared and cannulated in order to maintain artificial respiration (under room air, volume 1.5 ml/100g at a rate of 35 strokes/min, Harvard Rodent Ventilator 683, South Natick, MA, USA). The ECG (lead II) was continuously recorded and the arterial blood pressure was measured in the right *arteria carotis communis* (PowerLab 8 SP, ADInstrument). The left *vena jugularis* was cannulated for the drug administration and ouabain infusion. ORM-10962 (solved in DMSO: isotonic saline, 1:9) was administered in bolus 10 minutes before starting ouabain infusion. The control group received the solvent of the drug. Ouabain was continuously infused (at the rate of 10 μg/kg/min using an infusion pump (Terumo, TE-331), at the rate of 6ml/h/kg, intravenously.

In another series of experiments the suspension of ouabain induced arrhythmias by ORM-10962 was investigated. Ouabain (10 μg/kg/min) was infused for 16 min. ORM-10962 was administered intravenously after the termination of ouabain infusion and the ECG and blood pressure changes were recorded, as described above.

#### Myocardial ischaemia-reperfusion induced arrhythmias in rat

Male Sprague-Dawley rats were used. Coronary artery occlusion and reperfusion were performed as described previously [31,32]. During pentobarbitone anaesthesia (60 mg/kg intraperitoneally) the chest was opened between the fourth and fifth ribs and the heart was exposed. A loose loop of atraumatic silk (4/0 Mersilk black, W582, Ethicon) was placed around the left main coronary artery. After the surgery artificial respiration was started (at a rate of 60 strokes/min, volume 1.4 ml/min, Harvard Rodent Ventilator 683, South Natick, MA, USA). After 10 minutes normalization the coronary artery was occluded by tightening the loop for 6 minutes, followed by reperfusion for 5 minutes. Arterial blood pressure was measured via the right *arteria carotis comm*unis. The left *vena femoralis* was prepared and cannulated for drug administration. Solution of ORM-10962, as described previously, was administered 10 minutes before the coronary artery occlusion. ECG lead II was registered in both protocols. Heart rate and the time to the development of ventricular arrhythmias were measured on the ECG.

#### Myocardial ischaemia-reperfusion induced arrhythmias in guinea-pig

Male guinea pigs weighing 300-400g were used. Animals were anaesthetized with sodium-pentobarbital (300mg/kg, i.p.) and injected with heparin sodium (300 IU, ip.). Hearts were rapidly excised, mounted via the aorta on a Langendorff apparatus and retrogradely perfused with warm (37°C), modified Krebs–Henseleit bicarbonate (KHB) buffer at a constant pressure (80 mmHg). The KHB solution contained (in mmol/L): NaHCO_3_ 25; KCl 4.3; NaCl 118.5; MgSO_4_ 1.2; KH_2_PO_4_ 1.2; glucose 10; CaCl_2_ 1.8, having a pH of 7.4±0.05 when gassed with 95% O_2_ + 5% CO_2_. Ventricular fibrillations were induced by reperfusion following 20 minutes zero flow ischemia. The ECG was recorded using the WinWCP software (V4.9.1. Whole Cell Electrophysiology Analysis Program, John Dempster, University of Strathclyde, UK).

The animals (rats and guinea pigs) that during the *in vivo* experiments did not die from irreversible ventricular fibrillation were euthanized at the end of investigational protocol by overdosing anaesthetic drug (pentobarbitone).

#### Statistical analysis

Parameters were expressed as means ± SEM. and after analysis of variance (one-way ANOVA) groups were compared in pairs by means of the modified “t-statistics” of Wallenstein et al [33]. The survival rate was compared by using the Chi-square test.

### Materials

With the exception of ORM-10962 (from Orion Pharma, Espoo, Finland), nisoldipine (gift from Bayer AG, Leverkusen, Germany) and HMR-1556 (gift from Aventis Pharma, Frankfurt, Germany) all chemicals were purchased from Sigma-Aldrich Fine Chemicals (St. Louis, MO, USA) or Sequoia Research Products Ltd (Pangbourne, UK). ORM-10962 was dissolved in DMSO to yield a 1 mM stock solution. This stock solution was diluted to reach the desired final concentration (DMSO concentration not exceeding 0.1%) in the bath.

## Results

### Effects of ORM-10962 on the outward and inward NCX current

**Fig 2:**
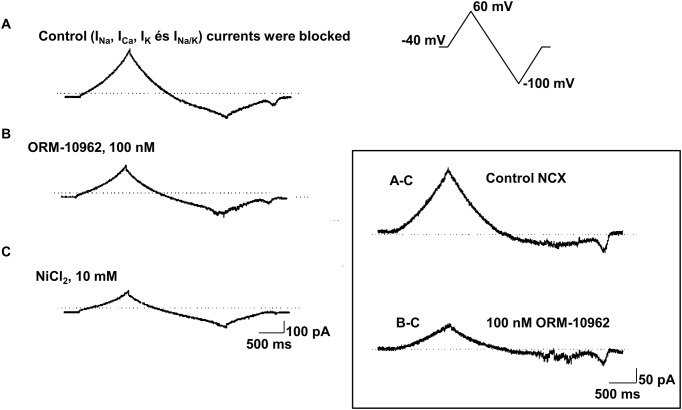
Determination of NCX current in dog ventricular myocytes. The protocol was adapted from Hobai *et al* [27]. **A:** Recording obtained with the voltage protocol shown in the inset in control conditions (current trace after blockade of Na^+^, Ca^2+^, K^+^ and Na^+^/K^+^ pump currents. **B:** The current trace after superfusion with 100 nM ORM-10962. **C:** The current trace at the end of the measurements after the application of 10 mM NiCl_2_. On the right the control NCX current is shown, which is obtained by subtracting trace **C** from trace **A**. The NCX current in the presence of 100 nM ORM-10962 is obtained by subtracting trace **C** from trace **B**. Note the difference in the intensity-time calibration in the left and right panels.

I_NCX_ was measured as Ni^2+^ sensitive current using voltage ramp waveforms for the command potential (Fig 2). The protocol adapted from Hobai *et al* [27] was described earlier in detail [21]. Current traces were recorded after blocking Na^+^, Ca^2+^, K^+^ and Na^+^/K^+^ pump currents. Then, ORM-10962 was applied and the current was recorded with the same voltage waveform. Finally, 10 mM NiCl_2_ was added to the bath solution for the complete blockade of the NCX current and this trace was subtracted from the control record and from the ORM-10962 record resulting in Ni^2+^ sensitive current traces in control conditions and in the presence of ORM-10962 (Fig 2).

**Fig 3:**
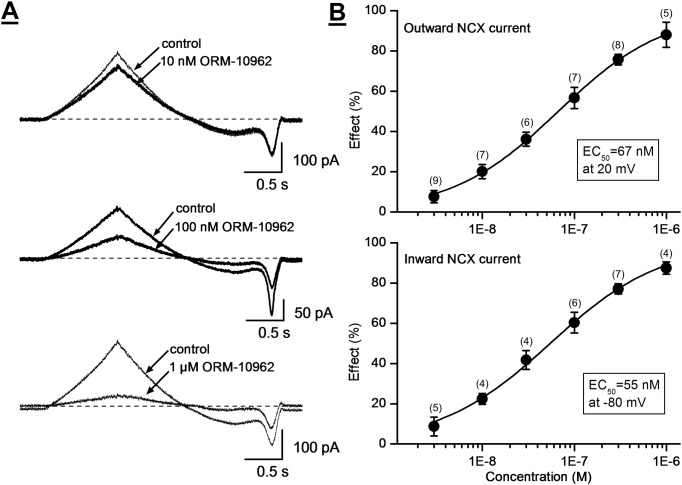
The concentration-dependent effect of ORM-10962 on the NCX current in dog ventricular myocytes. **Panel A**. Original Ni^2+^-sensitive (NCX) current traces before and after superfusion of the cells with ORM-10962 at concentrations of 10 nM, 100 nM and 1 μM. **Panel B.** The drug-response curve of ORM-10962 on the outward (top) and inward (bottom) NCX currents in dog ventricular myocytes is given at 20 mV and at -80 mV, respectively. Values are means ± SEM. Numbers in brackets show the number of experiments for each data point.

Fig 3 illustrates that ORM-10962 decreased the NCX current in a concentration-dependent manner with estimated IC_50_ values of 55 and 67 nM at -80 and at 20 mV, respectively (Fig 3). Under the conditions of our experiments, putative exchange currents reversed as expected about -30 mV, and the currents were decreased without a change of reversal potential by ORM-10962. We did not examine specifically whether the compound might inhibit preferentially the outward or inward current modes under conditions that would maximize the unidirectional exchanger operation.

### Selectivity of ORM-10962

**Fig 4:**
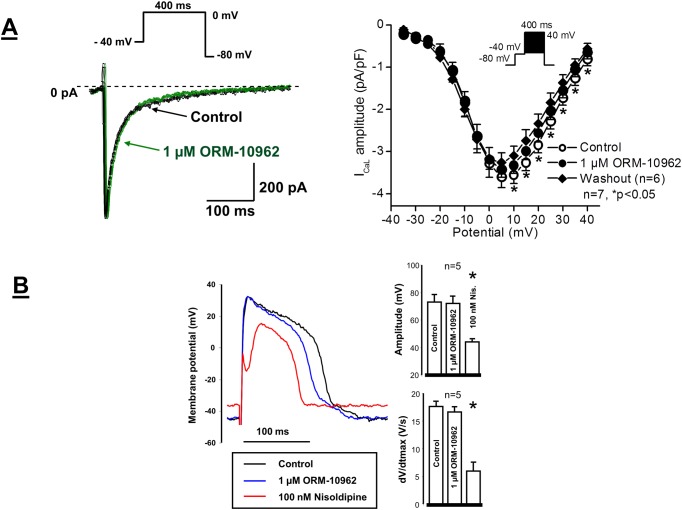
**Panel A.** Lack of effect of 1 μM ORM-10962 on the L-type I_Ca_ in dog ventricular myocytes. The panel illustrates original I_CaL_ current traces before and after application of 1 μM ORM-10962 (left panel) and the corresponding current-voltage relationship of I_CaL_ in the absence and presence of 1 μM ORM-10962, as well as after washout of ORM-10962 on an average of 7 myocytes. Values are means ± standard errors of the means, n = 7, *p<0.05. Insets show the applied voltage protocols. **Panel B.** Lack of significant effect of ORM-10962 on slow response action potentials in guinea-pig ventricular myocytes. Original slow action potential records under control conditions, in the presence of 1 μM ORM-10962 and after application of 100 nM nisoldipine (*left*). Bar diagrams (*right*) reveal the lack of effect of 1 μM ORM-10962 on the action potential amplitude and on the maximum rate of depolarization (dV/dt_max_), respectively. Values are means ± SEM, n = 5. *P<0.05.

The possible effect of ORM-10962 on the L-type calcium (I_CaL_) current was investigated in single dog ventricular myocytes. The current was activated by 400 ms depolarizing voltage pulses to potentials ranging from -35 mV to 55 mV. The holding potential was -80 mV. A prepulse to -40 mV served to inactivate sodium current. These experiments showed that ORM-10962 even at high (1 μM) concentration did not block I_CaL_ (Fig 4A). Though, after application of ORM-10962 a small but significant decrease of the current occurred, after wash out of the drug the current further decreased, which revealed that the slight reduction of the current amplitude in the presence of ORM-10962 was the consequence of the run-down phenomenon.

Since the I_CaL_ measurements in same experiments exhibited a small run-down in some voltage clamp experiments, the effect of ORM-10962 was also studied on slow response action potentials recorded from guinea-pig papillary muscles. As Fig 4B shows, ORM-10962 at 1 μM (a concentration about 20 fold higher than the IC_50_ for NCX inhibition) did not affect the amplitude (control: 73.2±0.7 mV, ORM-10962: 72.1±4.9 mV, n = 5, n.s.) or dV/dt_max_ (control: 17.6±0.7 V/s, ORM-10962: 16.6±0.9 V/s, n = 5, n.s.) of these slow response action potentials, suggesting the lack of effect of ORM-10962 on the L-type calcium current. In the same preparations the well-established calcium current blocker nisoldipine at 100 nM markedly reduced both the amplitude and dV/dt_max_ of the slow response action potentials (73.2±0.7 mV *vs* 44. 2±1.5 mV and 17.6±0.7 V/s *vs* 5.9±1.5 V/s, respectively; n = 5, p<0.05).

**Fig 5:**
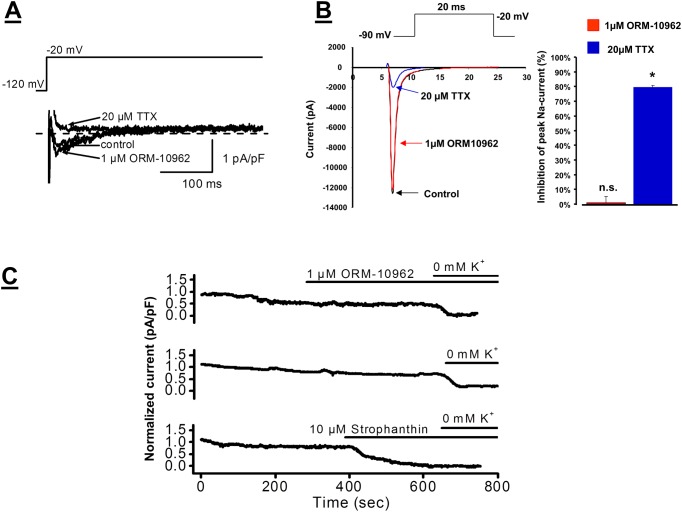
**Panel A**. Lack of effect of ORM-10962 on I_NaL_ current in dog ventricular myocytes. Original current traces show that 1 μM ORM-10962 does not influence I_NaL_, while 20 μM TTX blocks the current. The applied voltage protocol is illustrated on the top of the Figure. **Panel B.** Lack of effect of 1 μM ORM-10962 on peak I_Na_ measured in Nav1.5 expressed chinese hamster ovary (CHO) cells. The ORM-10962 did not influence the current (red line and bar), while 20μM TTX significantly reduced the current (blue line and bar). The applied voltage protocol is illustrated on the top of the Figure. **Panel C.** Lack of effect of ORM-10962 on Na^+^/K^+^ pump current. *Top trace* shows that 1 μM ORM-10962 does not influence I_p_, however, a slight gradual decrease of the current was detected. An original current record in the *middle trace* indicates that the slight gradual current decrease, similar to that shown in the *top trace*, was also observed without addition of ORM-10962. *Bottom trace*: Strophanthin (10 μM) completely blocks Na^+^/K^+^ pump current. I_p_ was defined as the difference between currents measured in 5 mM K^+^ and in 0 mM K^+^ containing solutions. The current traces were recorded at a steady potential of -30 mV.

The possible effect of ORM-10962 on I_NaL_ current was also studied in dog left ventricular myocytes. Fig 5A illustrates that addition of 1 μM ORM-10962 did not decrease the amplitude of I_NaL_, while 20 μM TTX completely blocked the current. The amplitude of the TTX sensitive I_NaL_ was -0.63±0.13 pA/pF in control conditions and -0.65±0.14 pA/pF after application of 1 μM ORM-10962 (n = 5, n.s.). The possible effect of ORM-10962 on peak I_NaL_ was measured in Nav1.5 expressing CHO cells (Fig 5B). Under control conditions the current was not significantly changed (-806.6±299.3 pA/pF *vs*. -782.7±257 pA/pF after 1 μM ORM-10962; Δ = 1.06±0.04%). The application of 20 μM TTX markedly reduced the current magnitude to 152.8±47.9* pA/pF (Δ = 79.6±0.01%; n = 10, p<0.05).

The Na^+^/K^+^ pump current (I_p_) was 0.46±0.10 pA/pF in control conditions and it decreased to 0.39±0.11 pA/pF (16.2%, n = 5, p<0.05) at the end of the 5–7 min incubation with 1 μM ORM-10962. During these measurements a gradual slight decrease of the steady-state current was observed (Fig 5C). Therefore, a separate set of experiments was performed when the same protocol was applied but ORM-10962 was not added to the bath solution. Measuring the steady-state current at the same time scale, comparing to the ORM-10962 experiments, a similar small current decrease was recorded (0.55±0.10 pA/pF *vs* 0.44±0.06 pA/pF, 21.5%, n = 3, n.s., Fig 5C). Therefore, it is concluded that ORM-10962 does not influence I_p_, the slight tendency of the current to decrease is not due to the effect of ORM-10962. In another experiment, 10 μM strophanthin, known to block Na^+^/K^+^ pump was applied. Strophanthin effectively diminished the current and 0 K^+^ solution failed to decrease the current further showing that the measured current is Na^+^/K^+^ pump current (Fig 5C).

**Fig 6:**
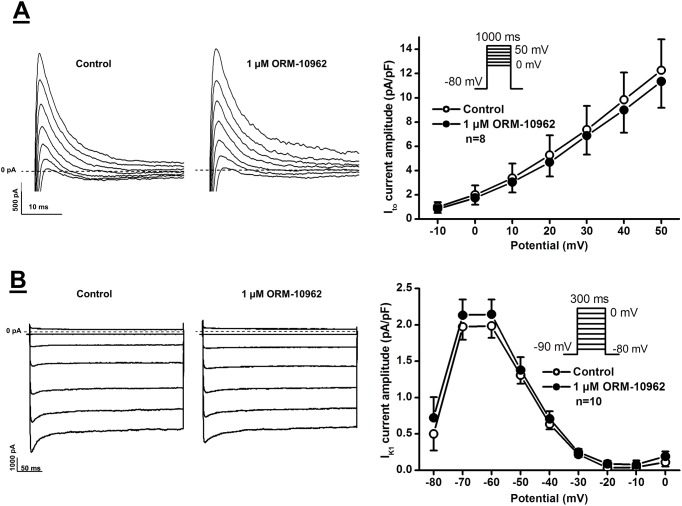
Lack of effect of ORM-10962 on the inward rectifier potassium and transient outward potassium currents (I_K1_ and I_to_) in dog ventricular myocytes. **Panels A and B.** Original I_K1_ (top) and I_to_ (bottom) current traces (left panels) recorded under control conditions and in the presence of 1 μM ORM-10962, and the corresponding I-V current-voltage relationships (right panels) before and after application of 1 μM ORM-10962, respectively. The insets in the I-V diagrams show the voltage protocol applied during measurements. Values represent means ± SEM, n = 8 and 10, respectively.

**Fig 7:**
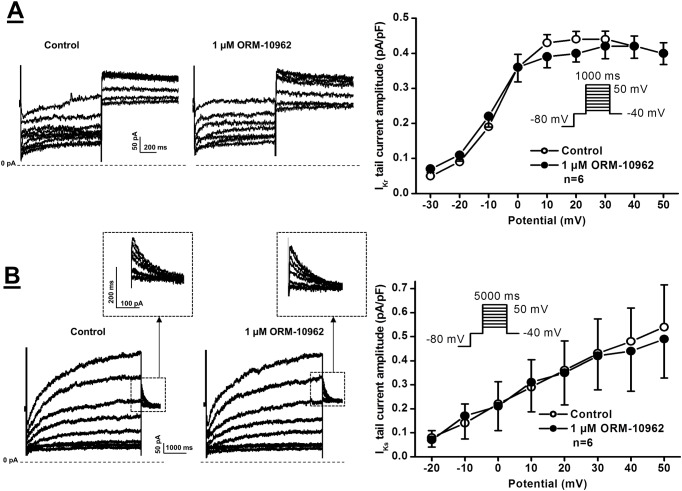
Lack of effect of ORM-10962 on the rapid and slow delayed rectifier outward potassium currents (I_Kr_ and I_Ks_) in dog ventricular myocytes. **Panels A and B.** Original I_Kr_ (top) and I_Ks_ (bottom) current traces (left panels) recorded under control conditions and in the presence of 1 μM ORM-10962, and the corresponding I-V current-voltage relationships (right panels) before and after application of 1 μM ORM-10962, respectively. The insets in the I-V diagrams show the voltage protocol applied during measurements. Values represent means ± SEM, n = 6 and 6, respectively.

The effect of ORM-10962 on the four main repolarizing potassium currents, I_K1_, I_to_, I_Kr_ and I_Ks_ currents was also investigated. Inward rectifier potassium current (I_K1_) was measured by determining the steady-state current at the end of 300 ms long pulses clamped to potentials ranging from -80 to 0 mV. The pulse frequency was 0.33 Hz (Fig 6A). Transient outward potassium current (I_to_) was activated by 1000 ms long depolarizing voltage pulses arising from the holding potential of -80 mV to test potentials gradually increasing up to 50 mV. The pulse frequency was 0.33 Hz (Fig 6B). The rapid and slow components of the delayed rectifier potassium currents (I_Kr_ and I_Ks_) were determined as tail current amplitudes at -40 mV after activating these currents by 1000 ms (I_Kr_) or 5000 ms (I_Ks_) long depolarizing voltage pulses of various test potentials ranging up to 50 mV. The pulse frequency was 0.05 Hz or 0.1 Hz, respectively. It was found that neither of these currents was significantly affected by 1 μM ORM-10962 (Figs 6 and 7).

### Effect of NCX inhibition on Ca^2+^ transient, cell shortening, and action potentials

The effect of 1 μM ORM-10962 on Ca^2+^ transients and cell shortening were studied simultaneously on isolated dog ventricular myocytes. The myocytes were field stimulated at 1 Hz. In these experiments 3 groups were separated.

**Fig 8:**
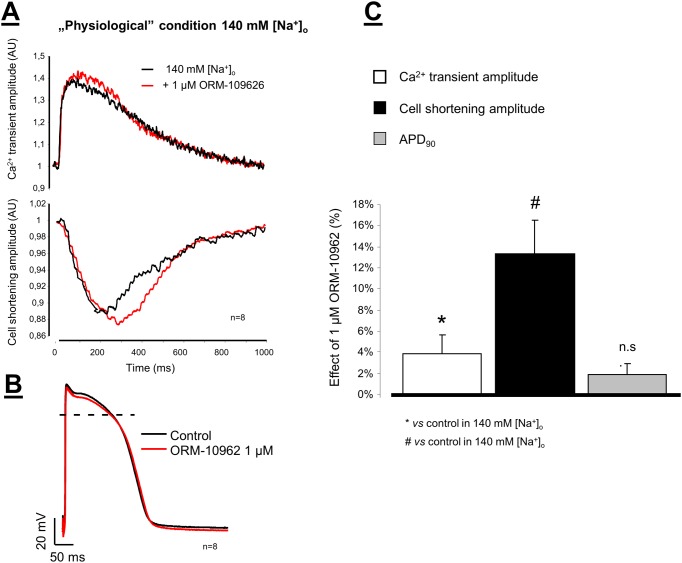
Ca^2+^ transients and cell shortening were simultaneously measured in isolated dog ventricular myocytes under normal conditions (**panel A**). The cells were loaded with Fluo-4AM fluorescent dye, and were field stimulated at 1 Hz. A small, but statistically significant increase in the transient and cell shortening magnitude was measured after application of 1 μM ORM-10962 (red line). The action potentials were measured by using the conventional microelectrode technique in dog right papillary muscles. In this case 1 μM ORM-10962 failed to influence the action potential characteristic (**Panel B**). The effects of ORM-10962 on Ca^2+^ -transient amplitude (white column), cell shortening (black column) and on APD_90_ (gray column) were compared in bar graph, depicted in **Panel C**.

In the *“physiological NCX operational mode”* we recorded the baseline Ca^2+^ transients and cell shortening in normal Tyrode’s solution then the perfusion was switched to normal Tyrode’s solution containing 1 μM ORM-10962 (Fig 8A and 8C). Under control condition the amplitude of Ca^2+^ transients and cell shortening increased only slightly but statistically significantly (Ca^2+^ transients: 15.03±2.16 AU *vs* 15.48±2.10* AU Δ = 3.80±1.49%*, n = 8, p<0.005; Cell shortening: -2.03±0.92 AU *vs*. -2.35±1.05* AU, Δ = 15.59±3.01%*, n = 8, p<0.05). The diastolic Ca^2+^ did not change significantly during the experiment. The characteristic of the action potential did not change after application of 1 μM ORM-10962 (APD_90_: 218.4±10.85 ms *vs* 222.5±9.66 ms; Δ = 1.86±1.55%; n = 8; Fig 8B and 8C).

**Fig 9:**
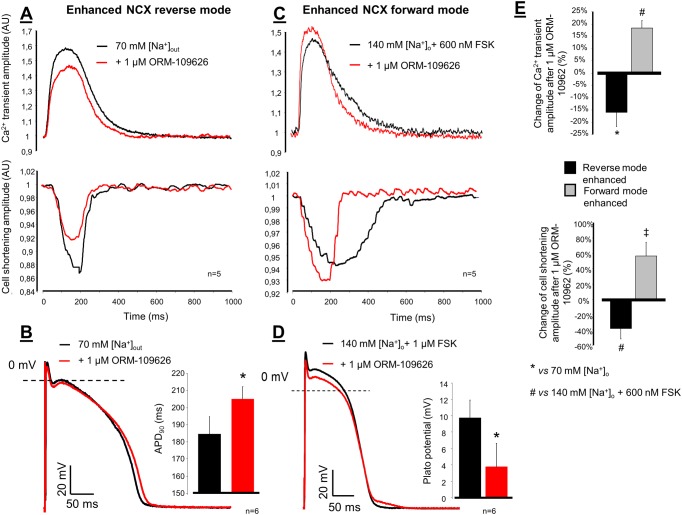
**Panel A** shows representative Ca^2+^ transient tracings and cell shortenings when NCX reverse mode was facilitated by reduction of the external NaCl to 70 mM. Under this setting, the application of 1 μM ORM-10962 significantly decreased the magnitude of Ca^2+^ transient (red line) and cell shortening (red line) compared to 70 mM NaCl containing Tyrode’s solution (black lines). In this case the APD_90_ of the action potential was slightly increased (**Panel B**). The forward mode of NCX was increased by 600 nM forskolin (**Panel C**). Under this setting 1 μM ORM-10962 further increased the amplitudes of the transient and cell shortening (red lines). The mid-plateau potential of the action potential significantly decreased (**Panel D**). The change of Ca^2+^ transient and cell shortening under reverse (black column) and forward mode (grey column) enhancement were depicted by bar graphs in **Panel E**.

In the *enhanced reverse NCX operational mode group*, the NaCl was reduced to 70 mM while the solution was supplemented with 70 mM choline-cloride. Under this condition the Ca^2+^ transients and cell shortening increased by the administration of low Na-Tyrode, and significantly decreased when 1 μM ORM-10962 was applied (Fig 9A). Ca^2+^ transients under control condition: 8.47±0.83 AU → low Na^+^: 11.53±2.50 AU → 1 μM ORM-10962: 9.46±1.98 AU*; n = 5, p<0.05. The transient amplitude decrease after ORM-10962 application was -16.07±5.77%*. The results of cell shortening measurements were the following: control: -3.80±1.4 AU → low Na^+^: -6.61±2.18 AU → 1 μM ORM-10962: -4.01±1.79 AU*; n = 5, p<0.05. The decrease of cell shortening was -38.12±16.45%* after ORM-10962 application. The diastolic Ca^2+^ significantly decreased after ORM-10962 application (*vs*. low Na^+^): 31.8±3.9 AU vs 28.6±2.9 AU; n = 5, p<0.05). Under this condition the APD_90_ of the action potential slightly but statistically significantly increased during ORM-10962 administration *vs*. 70 mM NaCl-contained Tyrode solution (184.5±10.4 ms *vs* 205.0±7.3 ms*; n = 6, p<0.05; Fig 9B).

In the *enhanced forward NCX operational mode group* the normal Tyrode solution was supplemented with 600 nM forskolin which increased the amplitude of transient and cell shortening. Application of 1 μM ORM-10962 increased further the amplitudes significantly (Fig 9C). Ca^2+^ transients under control condition: 15.89±1.82 AU → 600 nM forskolin: 13.72±1.50 AU → 1 μM ORM-10962: 16.21±1.80 AU*; n = 5, p<0.05. The increase of transient amplitude was 18.3±3.13% after ORM-10962 application. Cell shortening under control condition: -2.56±0.49 AU → 600 nM forskolin: -3.64±0.81 AU → 1 μM ORM-10962: -5.43±1.14 AU*; n = 5, p<0.05. The increase of cell shortening amplitude was 56.47±17.85% after ORM-10962 application. The diastolic Ca^2+^ did not change significantly during the experiment. The effect of these conditions on the transient and cell shortening amplitude is depicted in Fig 9E as bar graph. Under this condition the APD_90_ of the action potential did not change significantly, however the mid-plateau potential was significantly reduced (9.7±2.2 mV vs 3.7±2.9 mV*; n = 6, p<0.05; Fig 9D).

### Effect of NCX inhibition on *in vitro* triggered arrhythmias

**Fig 10:**
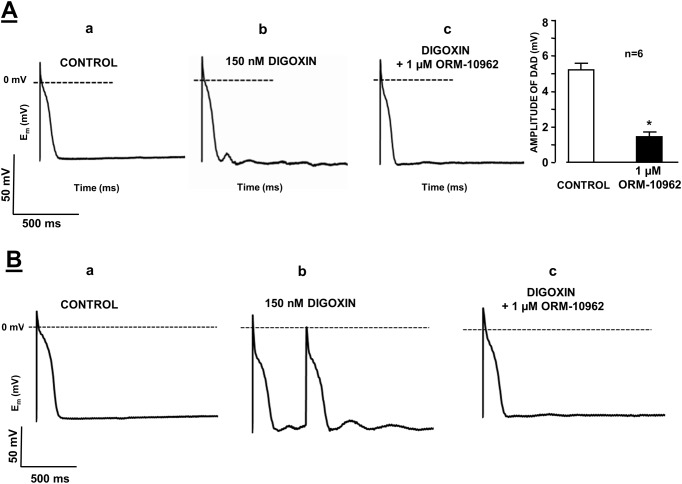
**Panel A.** The effect of 1 μM ORM-10962 on the delayed afterdepolarization (DAD) amplitude in dog right ventricular Purkinje fibres. DAD was evoked by a 40 stimulus train with a stimulation cycle length of 400 ms in the presence of 150 nM digoxin. Trace **a** is a control recording, trace **b** indicates the induction of DAD by 150 nM digoxin, and trace **c** demonstrates that 1 μM ORM-10962 almost completely abolished DAD. **Panel B.** Effect of ORM-10962 (1 μM) on digoxin-induced automaticity in dog right ventricular Purkinje fibres. Trace **a** is a control recording. Spontaneous activity was recorded after a 40 stimulus train with a stimulation cycle length of 400 ms in the presence of 150 nM digoxin (trace **b**). Application of 1 μM ORM-10962 in the presence of digoxin abolished the spontaneous activity (trace **c**).

The effects of ORM-10962 on delayed (DAD) afterdepolarizations were studied in dog cardiac Purkinje fibres by applying the conventional microelectrode technique. DAD was evoked in Purkinje fibre preparations superfused with 150 nM digoxin for 40 min (Fig 10A, left and mid panels). In these experiments, a train of 40 stimuli was applied with a cycle length of 400 ms in the train. The train was then followed by a stimulation-free period of 20 s to allow the observation of DAD formation. Following addition of 1 μM ORM-10962, the DAD amplitude was decreased (from 5.3±0.7 mV to 1.5±0.3 mV; n = 8, p<0.05; Fig 10A, right panel).

In six from 8 experiments, digoxin evoked a run of extra beats (cellular corresponding of the *in vivo* extrasystole) after the termination of the stimulus train, which could be successfully abolished by the application of 1 μM ORM-10962 (Fig 10B).

### Effect of NCX inhibition on in vivo triggered arrhythmias

#### Prevention of ouabain induced arrhythmias by ORM-10962 in guinea-pigs

**Fig 11:**
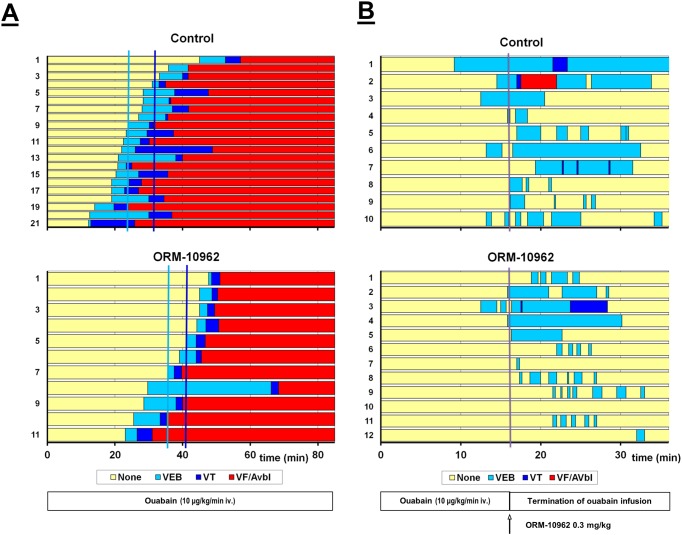
Effect of ORM-10962 (0.3 mg/kg) on ouabain (10 μg/kg/min i.*v*.) induced arrhythmias in anesthetized (pentobarbitone, 45 mg/kg *i*.*p*.) guinea-pigs. **Panel A**. ORM-10962 was applied as a pre-treatment 10 min before starting permanent ouabain infusion. Mean time to the development of ventricular arrhythmias is indicated on the Figure. **Panel B.** ORM-10962 was administered after the termination of 16 min ouabain infusion. In both **Panels** the ordinate indicates the numbering of the animals. There was a considerable antiarrhythmic effect; the duration of arrhythmic periods was shorter under the influence of ORM-10962.

Intravenous infusion of ouabain (10μg/kg/min) induced ventricular arrhythmias such as extrasystole, bigeminia, tachycardia, ventricular fibrillation and AV block in guinea-pigs. In the control group the first arrhythmias developed around the 24th minute. Seven-eight minutes later ventricular tachycardia appeared, that was followed by fibrillation around the 37^th^ minute after starting ouabain infusion. ORM-10962 (0.3 mg/kg) pre-treatment (*i*.*v*. 10 min before starting ouabain infusion) significantly delayed the development of ventricular extrasystoles (from 24±1.7 min in controls to 36.6±2.7 min in the presence of the drug, *p<0.05) or ventricular tachycardia (from 31.8±1.8 min in controls to 40.8±2.1 min in the presence of the drug, *p<0.05; Table 1 and Fig 11A).

**Table 1:** Effect of ORM-10962 (0.3 mg/kg) in ouabain (10 μg/kg/min *i*.*v*.) induced arrhythmias in anesthetized (pentobarbitone, 45 mg/kg *i*.*p*.) guinea-pigs. Time of the appearance of ouabain-induced arrhythmias in guinea-pigs.

Group	Dose (mg/kg)	N	VEB (min)	VT (min)	VF/AVbl(min)
Control	-	21	24.0±1.73	31.8±1.85	36.9±1.81
ORM-10962	0.3	11	36.6±2.64 *	40.8±2.17 *	42.4±2.1

Note: N: number of animals in the group; VEB: ventricular extrasystole, bigeminia; VT: ventricular tachycardia; VF/AVbl: ventricular fibrillation or complete atrioventricular block;

* P<0.05 (see Fig 11A).

There were no significant differences in the heart rate, systolic- and diastolic blood pressure (SP and DP) among the groups before the drug administration (-10 min). As a result of ouabain infusion heart rate and blood pressure increased. In the control group the last measured time-point was at the 19^th^ minute, because 24 minutes after the beginning of ouabain administration extrasystoles are already developed, followed by more serious ventricular tachycardia and fibrillation, until the death of the animal. ORM-10962 pre-treatment: i) did not induce significant effect on the basal heart rate and blood pressure; ii) delayed the blood pressure increasing effect of ouabain and the appearance of arrhythmias. Hence, the last measured pressure values before the beginning of the arrhythmias were at the 30^th^ minute (Table 2).

**Table 2:** Effect of ORM-10962 (0.3 mg/kg) and ouabain (10 μg/kg/min *i*.*v*.) in anesthetized (pentobarbitone, 45 mg/kg *i*.*p*.) guinea-pigs on the heart rate and blood pressure.

Group	Time (min)								
	-10	0	2	10	15	17	19	25	30
Control									
N	21	21	21	20	18	18	18		
HR	259±3.6	255±3.5	255±3.6	272±5.5	289±5	298±5.3	298±5.4		
SP	66±2.8	59±1.6	58±1.3	74±2.5	91±4.2	97±4.9	100±4.9		
DP	47±2.3	42±1.4	42±1.1	55±2.4	65±3.1	69±3.6	70±3.6		
ORM-10962									
n	11	11	11	11	10	11	11	9	7
HR	279±5.7	268±4.7	267±4	274±5	280±4.6	285±4.1	290±4.4	306±7.8	315±12.8
SP	56±3.2	51±3	50±2	64±2.8	72±3.4	84±2.3	90±1.9	104±2.9	117±6.2
DP	41±2.8	35±2.2	35±1.6	47±1.8	53±2.9	63±1.8	66±1.4	74±1.4	82±2.6

Note: N: number of the animals at the given time, HR: heart rate (1/min), SP: systolic blood pressure (mmHg), DP: diastolic blood pressure (mmHg), -10 min: baseline values before drug administration, 0 min: starting of the infusion of ouabain (see Fig 11A).

#### Suspension of ouabain induced arrhythmias by ORM-10962 in guinea-pigs

Intravenous infusion of ouabain (10μg/kg/min) did not produce severe arrhythmias during the 16 min infusion. Only 9 out of 22 animals developed sporadic ventricular eytrasystoles during this time. Termination of the infusion of ouabain, however, resulted in a further progression of arrhythmias. In the vehicle treated group, all of the animals developed some kind of arrhythmia, 3 animals showed ventricular tachycardia and 1 ventricular fibrillation (Table 3 and Fig 11/B). Heart rate and blood pressure could not be determined during this period due to frequent arrhythmias. After ORM-10962 treatment, the progression of ouabain induced arrhythmias was less expressed, only 1 animal developed ventricular tachycardia and no ventricular fibrillation occurred. The duration of the arrhythmic period was significantly shorter and the arrhythmias during this time were not continuous (Table 3 and Fig 11/B).

**Table 3:** Suspension of ouabain induced arrhythmias by ORM-10962, administered after the termination of ouabain infusion (10 μg/kg/min for 16 min) in anesthetized guinea pigs.

Group	Dose	N	Arrhythmic period	VEB	VT	VF/AVbl
	(mg/kg)		(min)	(min)	(min)	(min)
Control	-	10	14.22±2.29	10.5±2.5	0.3±0.2	0.5±0.5
ORM-10962	0.3	12	6.88±1.43 *	5.03±1.3	0.0	0.0

Note: N: total number of animals in the group; Arrhythmic period: Duration between the appearance of the first and the termination of the last arrhythmic event; VEB: ventricular extrasystole, bigeminia; VT: ventricular tachycardia; VF/AVbl: ventricular fibrillation or complete atrioventricular block;

* P<0.05 (see Fig 11B).

#### The lack of effect of ORM-10962 on coronary artery occlusion-reperfusion induced arrhythmias in rats

**Fig 12:**
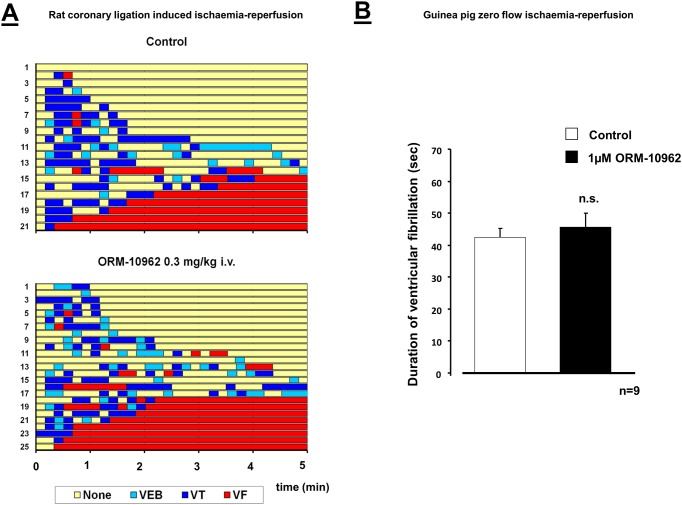
**Panel A**. Effect of ORM-10962 (0.3 mg/kg) in ischemia-reperfusion induced ventricular arrhythmias during reperfusion after 6 min coronary artery ligation in anesthetized (pentobarbitone, 60 mg/kg *i*.*p*.) rats. Time to the development of ventricular arrhythmias was measured on the ECG. The ordinates show the numbering of the animals. There was no significant change in the arrhythmias following administration of ORM-10962. **Panel B.** Effect of ORM-10962 (1 μM) in global ischemia-reperfusion induced ventricular fibrillation in isolated guinea pig hearts. ORM-10962 did not influence significantly the duration of ventricular fibrillation.

In control rats, after coronary artery ligation, arrhythmias appeared at around the fifth minute of the ischaemia. Coronary artery occlusion of 6 minutes rarely produced life-threatening arrhythmias, however, reperfusion after ischaemia rapidly induced severe arrhythmias, frequently leading to the death of the animals. Since the paucity of ischaemia induced arrhythmias, only the reperfusion-induced arrhythmias are presented. During reperfusion after 6 min ischemia 33% of the control animals died due to irreversible ventricular fibrillation. Other serious arrhythmias *e*.*g*. reversible fibrillation (48%) or tachycardia (62%) frequently occurred among the survivors. ORM-10962 pre-treatment did not result in any change in the development or the severity of these arrhythmias (Table 4 and Fig 12A).

**Table 4:** Effect of ORM-10962 (0.3 mg/kg) on ventricular arrhythmias induced by reperfusion after 6 min coronary artery ligation in anesthetized (60 mg/kg *i*.*p*.) rats.

Groups	N	Survived		Incidence of arrhythmias (n/%)				Arrhythmia score
		n	%	None	VF	VT	VEB	
Control	21	14	67	1/5	10/48	13/62	8/38	4.00±0.4
ORM-10962	25	17	68	0/0	15/60	14/56	14/56	4.12±0.35

Note: N: total number of animals; n (%): number and percentage of the animals showing the given response; None: animals without any arrhythmia; VF: ventricular fibrillation; VT: ventricular tachycardia; VEB: ventricular extrasystole and bigeminia (see Fig 12.)

There was no significant difference in heart rate, arterial systolic- and diastolic pressure among the groups before the coronary artery occlusion. As a result of the occlusion the blood pressure decreased in all groups. ORM-10962 pre-treatment did not influence significantly the blood pressure compared to the control group (Table 5).

**Table 5:** Effect of ORM-10962 (0.3 mg/kg) on the heart rate and blood pressure during ischaemia and reperfusion in anesthetized (60 mg/kg *i*.*p*.) rats.

		Time of ischaemia (min)						Time of reperfusion (min)		
Group	N	-10	0	1	3	5	N	1	3	5
Control										
HR	21	427±9.0	410±9.0	415±10.3	418±10.9	415±11.4	14	416±10.9	407±13.1	417±13.4
SP		108±2.3	107±2.3	78±2.6	81±3.4	84±3.6		89±8.5	95±7.6	94±6.6
DP		71±3.4	71±3.4	44±2.0	48±2.6	53±3.3		55±7.7	61±7.9	59±7.0
ORM-10962										
HR	25	411±8.5	397±8.2	410±9.6	404±9.4	401±10.7	17	387±14.5	400±10.1	384±15.0
SP		105±3.0	109±3.2	84±3.6	87±3.3	90±3.4		88±6.1	94±7.2	100±8.8
DP		73±3.3	77±3.7	54±2.8	58±2.7	61±3.0		56±5.2	64±6.9	69±7.5

Note: N: total number of animals, HR: heart rate (1/min), SP: systolic blood pressure (mmHg), DP: diastolic blood pressure (mmHg), -10 min: drug administration, 0 min: starting of the coronary artery occlusion (see Fig 12).

#### The lack of effect of ORM-10962 on zero flow ischaemia-reperfusion induced arrhythmias in guinea-pig Langendorff hearts

Zero flow ischemia-reperfusion induced ventricular fibrillations (VF) were measured in isolated guinea-pig hearts. Several seconds after the start of reperfusion ventricular fibrillations appeared in both groups. ORM-10962 (1 μM) pre-treatment did not cause a statistically significant difference in the duration of VF, compared to the control response (42.43± 2.79, *n* = 7 in controls *vs*. 45. 56± 4.44 in the presence of drug, *n* = 9), (Fig 12B).

## Discussion

The most important finding of this study is that the novel NCX inhibitor, ORM-10962, blocks the NCX current with high efficacy, at well below submicromolar concentrations (calculated EC_50_ values are 55/67 nM for inward/outward NCX currents, respectively). The inhibitory effect is also highly selective, since even at 20-fold higher concentration the drug had no apparent effect on any of the major transmembrane ion currents determining cardiac action potential, such as the L-type calcium current, peak and late sodium current, sodium-potassium pump and the main repolarizing potassium currents (I_Kr_, I_Ks_, I_to_ and I_K1_). Selective NCX inhibition is able to cause positive as well as negative inotropy depending on the actual experimental conditions by changing the operational mode of the NCX. Selective NCX blockade by ORM-10962 proved to be effective against DAD related arrhythmogenesis, since *in vitro* decreased the amplitudes of DADs evoked by digoxin in dog cardiac Purkinje fibres, and *in vivo* prevented ouabain induced cardiac arrhythmias in guinea pigs. In contrast, ORM-10962 had no apparent effect on ischaemia-reperfusion induced arrhythmogenesis generated by coronary artery (LAD) occlusion in rats and in Langendorff perfused guinea pig hearts.

Previous studies exploring selective NCX inhibition brought up two major points: the possible *positive inotropic effect* which could represent a novel strategy in the treatment of heart failure and the putative *antiarrhythmic effect*. Earlier, due to the lack of potent and selective NCX blockers these hypotheses could not be directly and properly tested. The first promising and credited “quasi-selective” NCX blockers were KB-R7943 and SEA-0400 [17,18,19,20]. Indeed, both SEA-0400 and KB-R7943 were shown to potently inhibit the NCX current at micromolar concentration range. In addition, KB-R7943 abolished experimental arrhythmias [4,15,18,19], while in another previous study we demonstrated that SEA-0400 effectively decreased the magnitude of both DADs and EADs in dog cardiac preparations [17]. Nonetheless, the proposed selectivity of both KB-R7943 and SEA-0400 were seriously questioned by studies revealing that at micromolar concentrations both blockers inhibit the L-type calcium current as well [4,15,18,19]. Therefore, in earlier studies the antiarrhythmic effects of NCX inhibition could not be safely separated from the antiarrhythmic effects of their inhibition of other transmembrane currents including I_CaL_. The positive inotropic effect was first tested by Hobai et al [34] in canine heart failure model applying the NCX inhibitory peptide XIP, which was able to restore the SR Ca^2+^ load and release. Ozdemir et al [35] also reported positive inotropic effect of SEA-0400 in a mouse heart failure model. The NCX inhibitor SN-6 has similar profile to that of SEA-0400, but it is more potent [36]. However, other study questioned its selectivity and claimed that SN-6 impairs contractility and Ca^2+^ handling [37]. The results of earlier NCX inhibitors were briefly summarized by Antoons et al. [20].

Recently, electrophysiological effects of another novel NCX inhibitor ORM-10103 were studied *in vitro* at the cellular level [21]. This compound also effectively blocked both forward and reverse NCX currents at concentrations close to 1 μM (estimated EC_50_ values are 780 nM and 960 nM, respectively). Contrary to ORM-10962, ORM-10103 had a small inhibitory effect on I_Kr_. Furthermore, it has not been tested in an *in vivo* arrhythmia model as yet.

In this previous study, inhibition of NCX by ORM-10103, another inhibitor, effectively prevented the strophantidine-induced Ca^2+^ accumulation and spontaneous diastolic Ca^2+^ releases when used as a pre-treatment. This was interpreted as a consequence of reverse mode inhibition [38]. Similarly, in the present study, ORM-10962 pre-treatment delayed the appearance of arrhythmias in *in vivo* guinea pig experiments (Fig 11A) that could be the consequence of reverse mode inhibition. In addition, when ORM-10962 was applied after intracellular Ca^2+^ overload and arrhythmias are already established (Figs 10 and 11B) the antiarrhythmic effect of NCX inhibition can be explained partially by forward mode inhibition resulting in less depolarization and thereby eliminating DAD’s in Purkinje fibres and suppressing arrhythmias in guinea-pig experiments.

### The selective NCX inhibition exerts slight positive inotropy under normal condition

Theoretical assumptions [5,7,12] as well as experimental results [38,39] suggest that in mammalian cardiomyocytes with long action potential plateau, the NCX mainly operates in forward mode extruding Ca^2+^ ions from the cell. Thus, theoretically, the decreased rate of Ca^2+^ extrusion may lead to net gain of the intracellular Ca^2+^ causing positive inotropy, while the reduced inward NCX current may shorten the action potentials [5,7,12]. In “physiological” NCX operational mode we found slightly increased Ca^2+^ transient amplitude and moderately enhanced cell shortening after selective NCX inhibition which supports the theory of slight Ca^2+^ accumulation. However, we failed to prove the change of action potential waveform (Fig 8). Our results may indicate an increase of the SR Ca^2+^ content without changes of the diastolic Ca^2+^ level. In our previous study, we characterized the NCX current during an action potential as an ORM-10103-sensitive current [38]. Since this current had relatively small amplitude we suggest that under normal circumstances the NCX current has marginal role in shaping the action potential. Furthermore, this small change may be compensated for other repolarizing currents. These results may also suggest that during a cardiac cycle similar extent of Ca^2+^ gain and loss should be expected by the operation of NCX. In other words, in physiological conditions the NCX inhibition is resulted in less intracellular Ca^2+^ and contractility changes than was previously believed. Therefore, the effect of NCX inhibition should be carefully studied in the future in different pathophysiological situations (ischaemia, heart failure, etc.) when NCX operational mode can be different from those in the normal “physiological” situation.

### Positive and negative inotropic effect of ORM depends on the actual operational mode of NCX

In contrast with the small positive inotropic effect of ORM-10962 found in this study (Fig 8), in our previous paper we described negative inotropic effect of selective NCX inhibition using ORM-10103 after 2 nM ATX application [38]. These disparate results suggested that NCX inhibition could equally lead to positive and negative inotropy depending on the actual NCX reversal potential. This study showed that the ATX-induced Na^+^ accumulation can markedly increase the ratio of the reverse mode—and thus NCX-mediated Ca^2+^ influx—which inhibition may have crucial role in the observed negative inotropy. In line with this, when [Na^+^]_o_ was reduced to 70 mM in this study, the reversal potential may shifted toward more negative range enhancing the contribution of the reverse mode to establish a new equilibrium. Thus, under this condition the NCX inhibition decreases the Ca^2+^ transient amplitude and cell shortening presumably *via* the suppression of the reverse mode.

In parallel experiments we intended to facilitate the NCX forward mode by applying forskolin to increase the adenylate-cyclase activity. We assume that the increase of the intracellular Ca^2+^ level without major rise of the intracellular Na^+^ may shift NCX reversal potential toward more positive values promoting forward mode activity. In this setting the observed positive inotropy may reflect further increase of SR Ca^2+^ content by reduction of the NCX mediated Ca^2+^ extrusion.

These data have led us to the conclusion that selective NCX inhibition may have negative inotropic and antiarrhythmic effect, when Ca^2+^ load is coupled with marked increase of intracellular Na^+^ and, this condition may facilitate the reverse mode activity of the NCX. This hypothesis was tested in dog Purkinje fibers where NCX inhibition almost completely abolished the digoxin induced DADs (see Fig 10). Under this setting the NCX inhibition may reduce the SR Ca^2+^ level primarily *via* the inhibition of reverse mode mediated Ca^2+^ influx, thus the forward mode may be indirectly reduced, following the decreasing intracellular Ca^2+^. Thus, we assume that the termination of DADs depicted in Fig 10, is a consequence of an indirect inhibition of the forward mode achieved by the decreased Ca^2+^ level caused by reverse mode inhibition, but some contribution of forward mode inhibition cannot be entirely ruled out.

It is worth mentioning that Ca^2+^ transients and cell shortening have somewhat different relaxation kinetics. The difference was not consistent and thereby statistically not significant. The exact reason for this discrepancy is not clear. Indirect Ca^2+^ sensitizing effect by ORM-10962 on the myofilaments cannot be ruled out. Also, Ca^2+^ transients reflect Ca^2+^ changes in the bulk of the cytosol which can differ from those of the subsarcolemmal space and in the close vicinity of the myofilaments.

### NCX inhibition is effective against ouabain arrhythmias but failed during ischemia-reperfusion induced arrhythmias

The selective NCX inhibition during *in vivo* ouabain-induced arrhythmias (Fig 11) in guinea pigs provided similar results compared with those we observed during digoxin-induced DADs in Purkinje fibers (Fig 10). We suggest that the underlying mechanism could be also similar: the antiarrhythmic effect may be based on the decreased intracellular Ca^2+^ level after inhibition of the reverse mode activity. Nevertheless, the role of some indirect NCX inhibition cannot be entirely excluded either. In contrast, ORM-10962 failed to reduce the arrhythmia incidence during ischaemia-reperfusion in rat *in vivo* (Fig 12A) and in guinea-pig *in vitro* (Fig 12B), even though several papers claimed the efficiency of NCX inhibition during ischaemia-reperfusion [15,40,41,42,43]. Interestingly, only some studies [44,45,46,47] were performed under *in vivo* conditions. Only one paper described antiarrhythmic effect of SEA0400 [46], the other studies reported failure of NCX inhibition (SEA-0400 and KB-R7943) to reduce arrhythmia incidence *in vivo*. Furthermore, in these studies SEA-0400 and KB-R7943 were used in such high concentration, which also inhibit the I_CaL_ current making the interpretation of the data more difficult. A previous study from our laboratory [48] reported lack of antiarrhythmic effect of ORM-10103 against ischaemia-reperfusion induced arrhythmias in Langendorff-perfused rats. The failure of NCX under this setting could be explained by the fact that the development of arrhythmias in ischaemia-reperfusion are rather complex phenomena involving several different mechanisms such as reentry, spontaneous automacy, DADs etc [49], and the NCX may not have important role in each mechanism. Furthermore, the lack of effect in rat as well as in guinea pigs may rule out the species dependent antiarrhythmic effect of selective NCX inhibition.

### Study limitations

We suggested that the antiarrhythmic action of NCX inhibition is mainly achieved by inhibition of the reverse mode and partially by suppression of the forward mode. This interpretation is based on speculations relating to our Ca^2+^ transient data and by considering our previous results [38]. However, the exact evaluation of the antiarrhythmic mechanism of NCX inhibition requires further experiments analyzing the effect of ORM-10962 on Ca^2+^ cycling and SR Ca^2+^ load in detail especially during pathological condition. Other possible limitation regarding the selectivity of ORM-10962 can be that we only studied its effects on transmembrane ion currents at fixed, relatively low intracellular Ca^2+^ concentrations. During the action potential, however, both intracellular Ca^2+^ and Na^+^ concentrations change markedly and dynamically. In theory, these changes can largely influence drug binding and consequently the efficacy of the compound on 2a variety of sarcolemmal ion channels. Another limitation concerns the possible role of NCX in arrhythmogenesis. These experiments were carried out in healthy, undiseased myocardium and in an acute ischemia model in the rat and in isolated guinea pig hearts. Arrhythmogenesis frequently occurs in diseased hearts (e.g. in heart failure, during chronic myocardial ischemia, etc.) in which calcium handling and NCX function may substantially differ from their state in healthy hearts, or following acute coronary ligation in hearts of otherwise healthy animals. Furthermore, species other than rat, having differing Ca^2+^-handling mechanisms, may respond differently to NCX inhibition in ischemia/reperfusion induced arrhythmias. Therefore, a number of further studies are needed to explore the potential therapeutic value of NCX inhibition in cardiac arrhythmogenesis.

## Conclusions and Clinical Implication

To our knowledge ORM-10962 is the first reported compound that can inhibit both forward and reverse NCX currents at well below submicromolar concentrations without affecting further essential sarcolemmal transport mechanisms. Selective NCX inhibition also exerts potent antiarrhythmic effect in digitalis induced arrhythmias. The previously reported antiarrhythmic effect of moderately selective compounds in ischemia/reperfusion induced arrhythmogenesis can be seriously questioned. While we suggest that selective NCX inhibition may have therapeutic implications by counteracting triggered automaticity, further detailed studies are needed before its cardioprotective role can be properly established.

## Abbreviations

APD: action potential duration
AU: arbitrary unit
cAMP: cyclic adenosine monophosphate
CHO: chinese hamster ovary cell line
CM: single ventricular cardiac myocyte
dV/dt_max_: maximum upstroke velocity of initial depolarization phase of action potential
DAD: delayed afterdepolarization
EAD: early afterdepolarization
I_CaL_: L-type calcium current
I_Na_: fast sodium current
I_NaL_: late sodium current
I_NCX_: sodium/calcium exchanger current
I_K1_: inward rectifier potassium current
I_Kr_: rapid component of the delayed rectifier potassium current
I_Ks_: slow component of the delayed rectifier potassium current
I_p_: Na^+^/K^+^ pump current
I_to_: transient outward potassium current
IR: ischemia-reperfusion
NCX: sodium/calcium exchanger
SAN: sinoatrial node
SP and DP: systolic- and diastolic blood pressure
VEB: ventricular extrasystole
VF/AVbl: ventricular fibrillation and/or atrio-ventricular block
VT: ventricular tachycardia
